# Radioprotective Effect of Melatonin on The Cervical Spinal Cord in Irradiated Rats

**Published:** 2013-02-20

**Authors:** Gholamhassan Haddadi, Alireza Shirazi, Zargham Sepehrizadeh, Seied Rabie Mahdavi, Maryam Haddadi

**Affiliations:** 1. Department of Medical Physics, Faculty of Medicine, Fassa University of Medical Sciences, Fassa, Iran; 2. Department of Medical Physics, Faculty of Medicine, Tehran University of Medical Sciences, Tehran, Iran; 3. Department of Pharmaceutical Biotechnology, Faculty of Pharmacy, Tehran University of Medical Sciences, Tehran, Iran; 4. Faculty of Medicine, Tehran University of Medical Sciences, Tehran, Iran

**Keywords:** VEGF, Spinal Cord, Melatonin

## Abstract

**Objective:**

It has been suggested that the vascular endothelial growth factor (VEGF) gene expression plays an important role in radiation-induced injury to the spinal cord. This study assesses the radioprotective effects of N-acetyl-5-methoxytryptamine (melatonin) through its modulation of VEGF expression after localized irradiation of the cervical spinal cord.

**Materials and Methods:**

In this experimental study, we divided 192 male rats into four groups: 1. control (n=48); 2. rats that received an intraperitoneal (IP) injection of melatonin (n=48); 3. rats that received an IP injection of melatonin 30 minutes prior to cervical spinal cord gamma irradiation [dose: 22 Gy; (n=48)]; and 4. rats that received an IP injection of vehicle prior to spinal cord irradiation (n=48). The changes in VEGF expression were assessed using real-time RT-PCR and enzyme-linked immunosorbent assays. Samples for light microscopy were stained with hematoxylin and eosin (H&E). The differences among the groups were analyzed using the analysis of variance (ANOVA) test followed by Tukey’s multiple comparisons test.

**Results:**

Up-regulation of VEGF expression was observed from 8 to 22 weeks after irradiation (p<0.05). Paralysis and other radiation-induced myelopathy manifestations developed within 22 weeks after irradiation. VEGF expression in the melatonin pre-treatment group significantly down-regulated in the 20^th^ and 22^nd^ weeks after irradiation compared to the radiation-only group.

**Conclusion:**

The results support the hypothesis that modulation of VEGF expression by melatonin administration may increase the survival rate of irradiated animals.

## Introduction

Radiation therapy plays an important role in the treatment of malignant head and neck tumors. However, radiation tolerance of the spinal cord is rather limited. Clinical data strongly suggests that a dose of 50 Gy given in 1.8-2.0 Gy fractions is associated with a 1% risk of spinal cord damage (1). Relatively high radiation doses are required to yield long-term local control in tumors with moderate radio sensitivities (2). The effect of radiation on a healthy spinal cord is one of the most important dose-limiting factors in radiation treatment. Radiation exposure to the spinal cord can result in myelopathy that often greatly impairs apatient’s quality of life (3). It has been shown that a single dose of 19-25 Gy to the spinal cord can cause limb paralysis with a latency of five months in rat models. The underlying mechanisms of this injury remain unclear, however there is an increasing amount of data indicating that the response of the central nervous system (CNS) after radiotherapy is a continuous, dynamic, and interactive process (4, 5).

Many authors have suggested that vascular endothelial growth factor (VEGF) plays a determining role in the disruption of the blood-spinal cord barrier (BSCB), in vascular alterations, and in the development of tissue necrosis (4). VEGF is a secreted, 46 kDa dimeric glycoprotein which acts as an endothelial cell specific mitogen, chemoattractant, and a vascular permeability factor (6). VEGF– induced increases in microvessel permeability and edema have been demonstrated in thenormal brain (7). Evidence shows that free radicals, such as radiation products, may play an important role in inducing the production of VEGF.

In the last decade, there have been reports on the radioprotective effects of N-acetyl-5-methoxytryptamine (melatonin), an endogenous compound primarily synthesized by the pineal gland in the human brain. Melatonin is a ubiquitously acting molecule with several functions. It is highly lipophilic and somewhat water-soluble. The widespread cellular distribution of melatonin may allow it to interact with all molecules, thereby reducing oxidative damage to molecules in both lipid and aqueous environments of the cell. It has been reported that melatonin directly scavenges highly toxic hydroxyl radicals both *in vitro* and *in vivo*, as well as several other reactive species such as singlet oxygen and peroxynitrite anions (8, 9). The free radicalscavenging capacity of melatonin is mediated by electron donation. The results of different studies indicate that both the acute and chronic toxicities of melatonin are extremely low (10).

Some *in vitro* studies have shown that melatonin can modulate the expression of VEGF that is induced by toxic agents (11). Other studies have shown that melatonin can decrease the permeability of the blood brain barrier (BBB) in cerebral ischemia (12). Furthermore, a previous study demonstrated the protective effect of melatonin on the early radiation-induced toxicity of the spinal cord (13, 14). In the present study, our goal was to assess whether melatonin administration could modulate VEGF expression after localized irradiation of the cervical spinal cord.

## Materials and Methods

### Chemicals

In this experimental study, melatonin acquired from Sigma-Aldrich was dissolved in ethanol and diluted with phosphate buffered saline (PBS) to a concentration of 10 mg/mL. All remaining reagents were obtained from either Sigma (St. Louis, MO, USA) or Merck (darmstadt, Germany).

### Experimental design

#### Animals

Adult male Wistar rats (180-220 g) were selected and housed in conventional rodent facilities. They were fed a standard diet of rodent chow and water and maintained at a constant temperature on a 12-hour light-dark cycle. The rats were divided into four groups. The first group (vehicle treated) served as the control. The second group (radiation treated) was treated with vehicle and exposed to radiation 30 minutes later. Group three (radiation + melatonin) was treated with an intraperitoneal (IP) injection of melatonin (100 mg/kg body weight) and exposed to radiation 30 minutes later in the same manner as the second group. The fourth group (melatonin-only) was also given an IP injection of melatonin (100 mg/kg body weight). Throughout the experiment, 5mg/kg of melatonin was administered daily to rats in groups three and four, and vehicle was administered daily to rats in groups one and two. The drug was administered between 4 and 5 pm. At this time of day, melatonin is considered to be at itslowest natural concentration in the blood. The dose of melatonin was selected based upon previous studies in the literature (14-16) and upon previous dose response studies.

#### Irradiation

Each animal was anesthetized with an IP injection of ketamine (60 mg/kg) and xylazine (20 mg/ kg) and then placed in the prone position. Rats in groups 2 and 3 were irradiated with a gamma beam of the Cobalt-60 teletherapy unit (Theratron 760-C) to the 1.8 cm cervical segment of the spinal cord (C1-T2). A single dose of 22 Gy at a dose rate of 1.8 Gy/minute and source skin distance of 79.5 cm was administered to a depth of 0.5 cm based on lateral simulation radiographs. This dose has been proposed to be the effective dose for white matter necrosis and limb paralysis after 20 weeks of irradiation (17). Sham irradiation was also performed for control and melatonin-only groups where the rats were anesthetized, but not irradiated.

#### Sample preparations

The animals were anesthetized (ketamine and xylazine injections) at 4 and 24 hours, and 1, 3, 8, 16, 20 and 22 weeks followingradiation treatment. For each time point, we used five rats. Tissue sampling was done using a posterior approach to the cervical spinal cord. A total of 1cm of spinal cord was dissected and used for histopathological and real time RT-PCR studies. The spinal cord was embedded in GITC (6 M), whichinactivates enzymes andcreates RNasefree conditions. It was also homogenized using a Heidolf homogenizer. All samples were stored at -70℃ until needed.

#### RNA isolation and real time RT-PCR

Total RNA from the spinal cord was isolated using a High Pure RNA Extraction Kit (Roche) following the manufacturer’s instructions. The quality of extracted RNA was verifiedby using a denaturing agarose gel and quantified with a Biophotometer (Eppendorf, Canada). After quantifying the RNA, 1 µg of the total RNA was denatured at 65℃ for 5 minutes. The tube was then placed on ice for 2 minutes, and reverse transcription was carried out in a solution that contained 1 µL expand reverse transcriptase (Roche), 4 µL buffer, 1 µL dNTPs (10 mM), 1 µL DTT, and 1 µL oligo (dT)_15_ (20 pmol) fora total volume of 20 µL at 42℃ for 60 minutes. Separate PCR reactions were performed for the amplification of cDNA for β-actin (the internal standard) and VEGF. Specific primers and SYBR Green PCR Mix were purchased from the Superarray Company. PCR reactions were carried out in a reaction volume of 25 µLthat consisted of 5 µL cDNA, 20 pmol of each primer, and 12.5 µL PCR-mix. Thermal cycling was initiated with an initial denaturation step at 94℃ for 3 minutes followed by the thermal profile of 94℃ (20 seconds) + 55℃ (30 seconds) + 72℃ (40 seconds) for 40 cycles in a Stratagene real-time PCR system. A suitable threshold was applied to the amplification plots and the resultant Ct values (threshold cycles) were used for relative quantification. The Ct values of VEGF were normalized according to the Ct values of β-actin and the results compared with those of the control group using the 2-ΔΔct method (18). The relative amount of VEGF compared to the control group was determined by dividing the normalized amount of VEGF in each sample by the amount of VEGF in the control samples at that time point.

#### Biochemical procedure for VEGF protein assay

Tissue samples were homogenized in 400 ml PBS. The homogenates were centrifuged at 15000 rpm for 30 minutes at 41℃. The supernatant was collected and stored at -78℃ until further analysis. The VEGF concentration was determined using an enzyme linked immunosorbent assay (ELISA) kit specific for VEGF (Biosource, Camarillo, CA, USA) according to the manufacturer’s specifications. The VEGF level of each sample was evaluated as the VEGF protein concentration (mg/l) divided by the total protein concentration (g/l) dissolved in a sodium dodecyl sulfate solution. Results were expressed as picograms per milligram of tissue protein (pg/mg protein).

#### Histopathological studies

The samples for light microscopy were immediately immersed in an appropriate fixative and stained with hematoxylineand eosin (H&E). Samples were prepared for transmission electron microscopy (TEM) toevaluate morphological changes in the endothelial cells. TEM preparation consisted of fixation, embedding, semi-thin formation and toluidine blue staining. Examination was performed under appropriate magnification to observe morphological changes in endothelial cells. Histopathological damage was scored on a scale of 0-3 (0-none, 1-mild, 2-moderate, and 3-severe). Images of spinal cord sections were examined by a histopathologist for changes in white matter stroma and vascular alterations.

#### Clinical responses survey

In another set of experiments, 20 rats from the control, irradiated, and irradiated plus melatonin groups were followed for 50 weeks after they received 22 Gy irradiation to the cervical spinal cord. The rats were monitored every other day for the development of paralysis in the hind and fore limbs (clinical endpoint). As soon as the neurological signs were evident, the rats were sacrificed under ketamine and xylazine injection, and specimens of the cervical spinal cord were prepared for histopathological analyses. The clinical diagnosis was thus verified histologically by identifying lesions consistent with radiation myelopathy. All procedures in this study were in accordance with the Guidelines for the Care and Use of Laboratory Animals as adopted by the Ethics Committee of the School of Medicine at Tehran University of Medical Sciences.

#### Statistical analysis

The data are presented as mean ± SEM. The differences among the groups were analyzed using the analysis of variance (ANOVA) test followed by Tukey’s multiple comparisons test. Survival data were analyzed in an actuarial fashion using a Kaplan-Meier analysis and compared with the log-rank test. P<0.05 was considered significant.

## Results

### Change in VEGF gene expression

**Fig 1:**
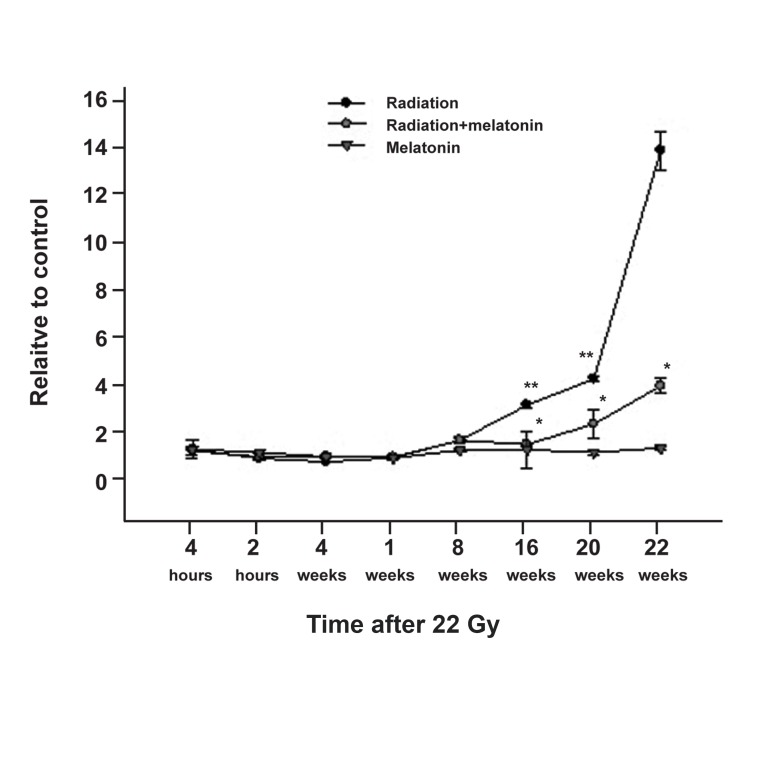
Profile of VEGF gene expression changes as a function of time after 22 Gy gamma irradiation-only and melatonin administration. VEGF gene expression increased rapidly in the weeks immediately preceding paralysis (*p<0.05 radiation vs. control groups). Melatonin administration modulates the expression of this gene (**p<0.05 radiation + melatonin vs. radiation groups). Data are mean ± SEM.

After a single dose of 22 Gy, VEGF gene expression was measured in the irradiation group, melatonin pretreatment + irradiation group, and melatonin treatment-only group. Finally, expression was defined proportionate with the age-matched controls after 4 and 24 hours, and 1, 3, 8, 16, 20 and 22 weeks after irradiation (Fig 1). Within 8 weeks after irradiation, VEGF gene expression up-regulated 1.5-fold in the irradiated group compared to the control group. VEGF expression in the irradiated group increased over time when the interval after radiation exposure was extended to 22 weeks. Within 16 weeks after irradiation, the up-regulation of VEGF gene expression in the irradiated group was 3-fold, and within 20 weeks it was 4-fold, compared to the control group (p<0.05). However, VEGF gene expression increased rapidly in the 22^nd^ week after irradiation and reached 14-fold in the irradiated group compared to the control group (p<0.01). At this point the irradiated animals began to shown signs of paralysis. VEGF gene expression in the melatonin pretreated group was significantly down-regulated at 16, 20, and 22 weeks after irradiation (p<0.05) compared to the radiation-only group. There was no significant difference between the control and melatonin-only groups.

**Fig 2:**
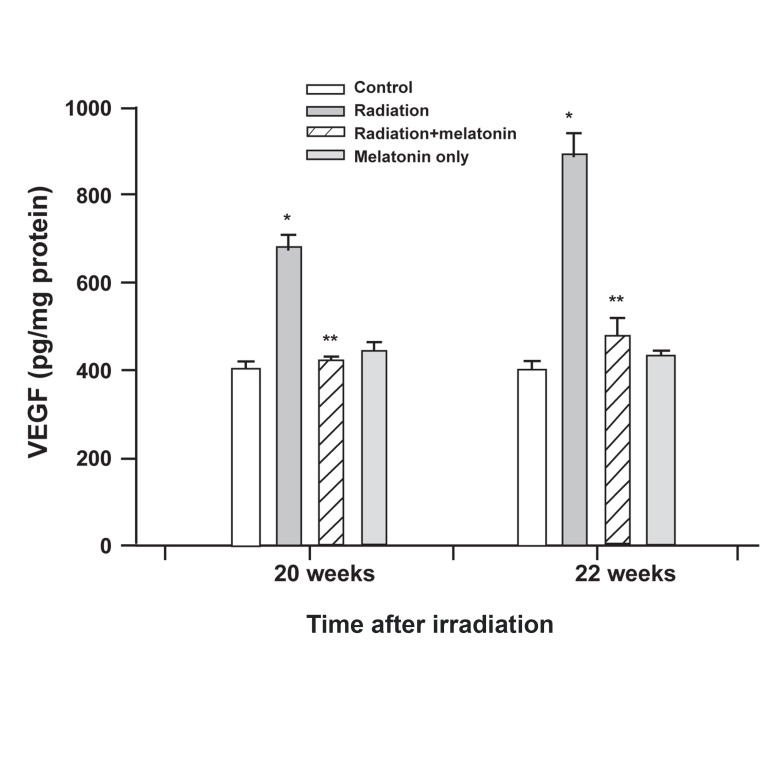
Effect of pre-treatment with melatonin on VEGF protein levels (pg/mg protein) at 20 and 22 weeks after irradiation. VEGF levels of the irradiated groups are significantly higher than the control groups (*p<0.05 radiation vs. control groups). Melatonin significantly reduced VEGF levels in the spinal cords of rats subjected to irradiation (**p<0.05 radiation + melatonin vs. radiation groups). Data are the mean ± SEM of six rats.

We next assessed whether the influence of melatonin on VEGF mRNA levels also corresponded to a decreased production of VEGF protein. Within 22 weeks after radiotherapy VEGF protein levels in the samples were measured by ELISA. At these time points VEGF levels in the spinal cord tissue samples were found to be significantly higher in the irradiation group than in the control group (p<0.01). The levels of VEGF protein were notably lowerin the radiation + melatonin group compared with that of the radiation- only group (p<0.05). No significant differences in VEGF protein levels were seen in the control and melatonin-only groups. The VEGF protein levels for all experimental groups are shown in figure 2.

### Histopathological assay

**Fig 3:**
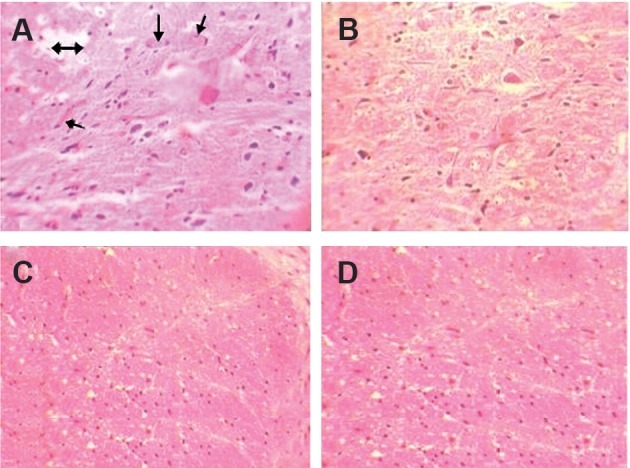
Histopathological effect of 22 Gy gamma radiation and protection by melatonin 22 weeks after irradiation: A. Vasodilation and congestion (arrows), and cavitation (double arrow) of white matter in the irradiated spinal cord. B. Prominent reduction in vasodilation, congestion, and cavitation in the melatonin treatment group. C, D. No evidence of any vascular abnormality in the control and melatonin groups (H&E staining).

H&E-stained spinal cord sections from groups sacrificed at early time points after irradiation showed no marked histopathological changes. Pathological changes in both vascular and white matter parenchyma of the irradiated groups began at 16 weeks after irradiation. Severe vessel dilation and cavitation was observed at 22 weeks after irradiation (Fig 3). There were statistically significant differences in the total effects of radiation in these irradiated groups compared to the control groups. The differences in vascular changes between the control and melatonin pretreated groups were not significant.

**Fig 4:**
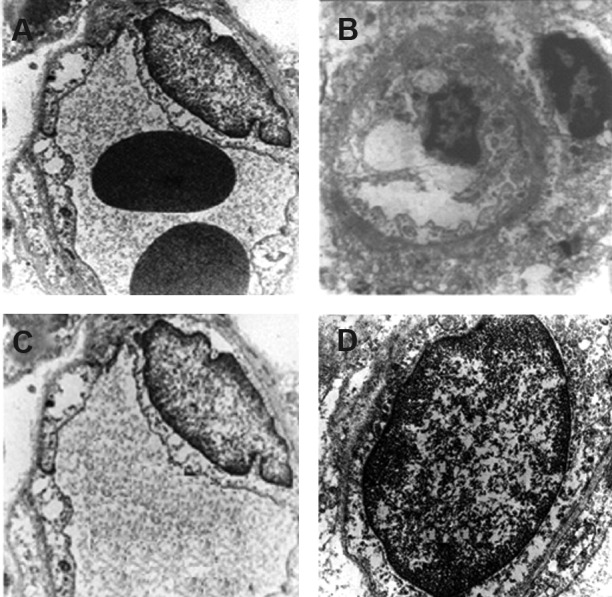
Transmission electron microscope (TEM) images of rat cervical spinal cord white mattercapillary vessel endothelial cell: A. Endothelial cells from a capillary of the melatonin + irradiated group of rats are shown. The nucleus of the cell, endothelial cell membrane, two RBCs within thelumen, and some of the cellular organelles including the endoplasmic reticulum and normal appearing mitochondria.B. 22 weeks after irradiation, endothelial cell withprominent, condensed nucleus, and irregular, deformed cytoplasmic organelles areseen. C, D. Normal structure endothelial cells from a capillary of control and melatonin groups.

We have used TEM because the endothelial cells of the vessels are a target for VEGF andto examine the architectural alterations induced by the changes in VEGF levels on endothelial cells. Sections from rats sacrificed 22 weeks after irradiation were used in the TEM study. Control and melatonin-only groups showed normal ultra-structural architecture in the vascular endothelial cells of the white matter. Irregularities in the thickness of the endothelial cell membranes with an increased number of intracellular organelles were noted in the irradiated group at 22 weeks after irradiation. There were no irregularities noted in the endothelial cell membrane in tissue samples from the melatonin + radiation group (Fig 4).

### Frequency and onset of myelopathy

**Fig 5:**
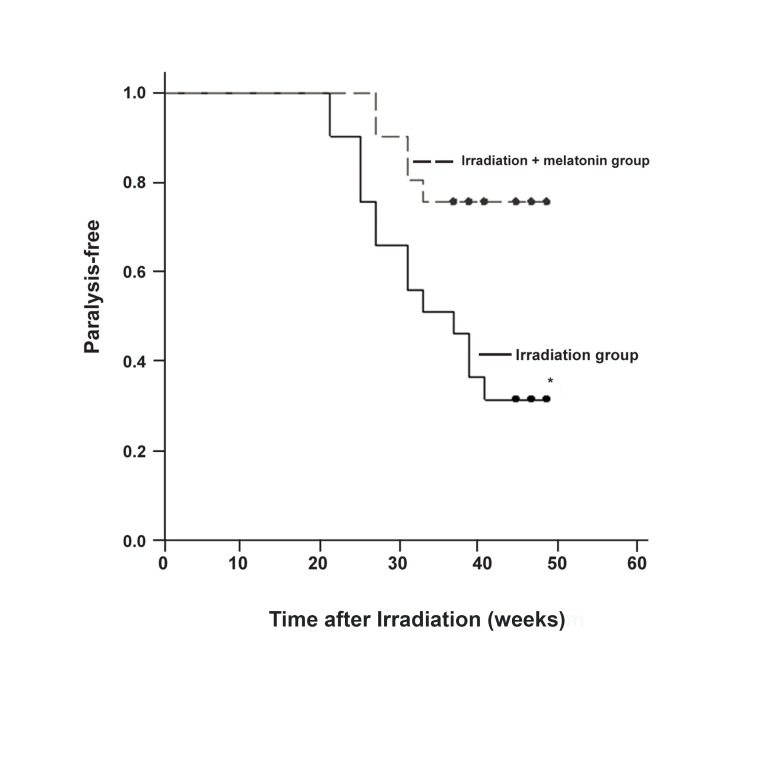
Kaplan-Meier curve of paralysis due to myelopathy for the two groups. The irradiation + melatonin group did considerably better than the irradiation group in terms of paralysis (*p<0.05 vs. irradiation group).

 Irradiated rats showed a shorter latency period for radiation-induced hindlimb paralysis and weakness compared to radiation + melatonin treated rats. The mean latency period (LP50) for the irradiated group was 36.8 ± 2.3 weeksand for the irradiated + melatonin groupit was 45.2 ± 2.1 weeks. There were significant differences in the incidence of radiation myelopathy (RM) between the irradiated and irradiated + melatonin groups (p<0.01, Fig 5).

## Discussion

Myelopathy can be a serious complication of spinal cord irradiation. Studies show that endothelial cell damage from radiation is one of the mechanisms involved in radiation-induced myelopathy (3). Although the exact molecular mechanisms leading to this delayed injury are not fully understood, it has been proposed that VEGF as an important determinant of microvascular permeability plays a role (20). Melatonin has been reported to have radioprotective effects in addition to its known hormonal activities (8). This agent crosses the BBB, and is a highly effective antioxidant in the brain (21). Many reports assert that melatonin canmodulate the expression of VEGF *in vitro* (11), thus we have assumed that melatonin may have radioprotective effects via *in vivo* VEGF down-regulation.

In this study, we outlined the variations in the expression profile of VEGF within the rat cervical spinal cord from 4 hours to 22 weeks after 22 Gy irradiation. The effects of melatonin on this profile were then investigated.

The results indicated that VEGF expression was not an early response to irradiation, but rather a delayed reaction. The delayed increase in VEGF expression occurred 16 weeks after irradiation and increased over time. Comparable results were reported by Nordal,who demonstrated increased expression of VEGF using immunohistochemistry and in situ hybridization (20). VEGF over-expression has been shown in other CNS injuries due to focal ischemia (21). Histopathological studies revealed changes in the vasculature of white matter that were associated with VEGF expression. There was a steep correlation between VEGF protein expression and vascular damage. Ultrastructural studies of the endothelium have also shown delayed changes in the cell membrane including destruction of the cell wall, thickening of the basal membrane, and detachment of the endothelium. In irradiated rat spinal cord, endothelial cell death or damage leads to blood-spinal cord disruption, vasogenic edema, vascular compromise, and tissue hypoxia. Hypoxiainduces VEGF expression in reactive astrocytes, which in turn leads to further increases in vascular permeability and disruption of the BSCB. Theabrupt release of excessive amounts of intracellular and extracellular oxygenfree radicals may initiate many chain reactions, leading to the release ofcytokines and VEGF. This may trigger an avalanche effect, resulting in white matter necrosis (22, 23). In our study, the free radical scavenging properties of melatonin may prevent these chain reactions. Recently, some studies have shown that melatonin suppresses the VEGF level *in vitro* and *in vivo* (11, 24).

The findings showed that prophylactic administration of melatonin significantly increased the latency period and delayed the onset of paralysis in irradiated animals. Prophylactic melatonin administration also reduced the RM incidence in these animals. These findings wereconsistent with a study by Blickenstaff et al. who reported that when mice were pre-treated with melatonin, 43% of the irradiated ones survived for at least 30 days after exposure to a lethal dose of ionizing radiation (25). Furthermore, Vijalaxmi et al. (26) observed that pretreatment with melatonin at a dose of 125 mg/kg body weight increased survival by up to 60%. The results of both the VEGF expression assays and the histological studies in the present study demonstrated that in the treatment group, melatonin decreased expression of VEGF in the spinal cord and considerably reduced the rate of paralysis.

Most likely regulation of VEGF is a complicated process; in addition to the anti-oxidative effects of melatonin, other transcriptional factors may be involved its regulation of VEGF expression. Reiter noted that "melatonin does not function exclusively as a free radical scavenger and antioxidant, but may have other functions which help cells and organisms to cope with metabolic disasters". For example, melatonin can influence NFkB, a multifunctional transcription factor that is capable of influencing a variety of genes (27).

## Conclusion

Although more investigation in this field is needed to better clarify the mechanisms of melatonin in VEGF down-regulation and its relation to histopathological alterations, the data of our study suggest that melatonin administration may be useful in late radiation-induced toxicity via VEGF downregulation. The precise role of melatonin in VEGF down-regulation and neuroprotection after irradiation remains to be determined.
